# ACE2 expression and sex disparity in COVID-19

**DOI:** 10.1038/s41420-020-0276-1

**Published:** 2020-05-26

**Authors:** Maria Cristina Gagliardi, Paolo Tieri, Elena Ortona, Anna Ruggieri

**Affiliations:** 1 Center for Gender Specific Medicine, Istituto Superiore di Sanità, Rome, Italy; 2 CNR National Research Council, IAC Institute for Applied Computing, Rome, Italy

Coronavirus disease 2019 (COVID-19) death rate differs depending on sex: in Chinese confirmed cases, while the infection rate among men and women is similar, the death rate among men is 4.7% compared with 2.8% for women^1^. Italian data are similar as the reported death rate in men is significantly higher than that in women, 16.6% vs. 9.1%, respectively. Moreover, preliminary data from Italian epidemics suggest also a significant sex difference in infection rate, being 52.5% in women and 47.5% in men (Integrated Surveillance on COVID19 epidemic in Italy published by Italian Institute of Health, April 28th 2020, https://www.epicentro.iss.it/coronavirus/bollettino/Bollettino-sorveglianza-integrata-COVID-19_28-aprile_2020.pdf).

So far, the mechanisms underlying the observed gender bias are not disclosed; however, some hypotheses can be put forward on the basis of current knowledge on gender differences in respiratory viral diseases.

The sex different lifestyles, such as smoking addiction that is prevalent in men than in women, is considered one of the potential risk factor for developing pneumonia consequent to COVID-19^2^. In addition, it is known that, in general, innate and immune responses are more intense and stronger in females than in males^3^. This can provide women with a more effective weapon to fight new and infective pathogens, favouring viral clearance.

However, further factors could be taken into account in order to explain the sex bias in COVID-19 death rates. In particular, the human angiotensin-converting enzyme 2 (ACE2), an essential enzyme of the renin–angiotensin system (RAS), is the functional receptor for the severe acute respiratory syndrome coronavirus (SARS-CoV) as well as for the recently identified SARS-CoV-2^4,5^. It has been shown that ACE2 plays a protective role in chronic pathologies, like hypertension, cardiovascular diseases, and acute respiratory distress syndrome, that are the comorbidities representing the risk of worse prognosis in COVID-19. The protective role of ACE2 has been evidenced by studies in mice models, showing more severe lung failure upon ACE2 down-regulation^6^.

Intriguingly, infection with SARS-CoV induces ACE2 down-regulation through binding of the viral Spike protein to ACE2, thus reducing ACE2 expression in the lung and igniting acute respiratory failure^6^. Since COVID-19 and SARS patients share similar acute respiratory distress syndrome and a similar gender bias in disease susceptibility and case fatality rates, it is reasonable to believe that they share similar pathogenic mechanisms^7^.

Estrogen, the primary female sex hormone, has been observed to play a protective role in SARS not only by activating immune response but also suppressing directly SARS-CoV replication^8^. To note, estrogen inhibits the activity or expression of different components of the renin–angiotensin system. In particular, estrogen is able to upregulate the expression of ACE2^9^.

Furthermore, the gene encoding ACE2 is located on the X chromosome, in sites commonly escaping the inactivation of one X chromosome in mammalian XX cells (XCI), a mechanism that determine the X chromosome transcriptional silencing and avoids redundant gene expression in female cells. However, the silencing is not complete but about 10% of the genes escape the inactivation; as a consequence XX cells over-express genes located in XCI sites, like ACE2^10^.

Consequently, hormonal and genetic factors could lead to ACE2 over-expression in female sex. These insights could, at least partially, account for the better outcome and the lower death rate in female SARS-CoV-2 patients with respect to males. All in all, further studies appear as mandatory to evaluate: (i) whether women who use hormone replacement therapy (HRT) are better protected from respiratory failure than women who do not use HRT; (ii) whether estrogenic agonists, increasing ACE2 expression levels, could represent promising tools to fight the COVID-19 outbreak. Notwithstanding, the study of the role of XCI escaping genes, and of their regulators, could represent a major challenge to understand the sex-specific pathogenic determinants of COVID-19 disease progression.
